# Straightforward thiol-mediated protein labelling with DTPA: Synthesis of a highly active ^111^In-annexin A5-DTPA tracer

**DOI:** 10.1186/2191-219X-2-17

**Published:** 2012-04-27

**Authors:** Harald Kratz, Akvile Haeckel, Roger Michel, Lena Schönzart, Uli Hanisch, Bernd Hamm, Eyk Schellenberger

**Affiliations:** 1 Department of Radiology, Charité - Universitätsmedizin Berlin, Charitéplatz 1, 10117, Berlin, Germany; 2 AnaKat – Institut für Biotechnologie GmbH, Robert-Koch-Platz 4, 10115, Berlin, Germany

**Keywords:** Annexin A5, Apoptosis imaging, PET, SPECT, DTPA, Indium

## Abstract

**Background:**

Annexin A5 (anxA5) has been found useful for molecular imaging of apoptosis and other biological processes.

**Methods:**

Here, we report an optimised two-step synthesis of annexin A5-diethylene triamine pentaacetic acid (DTPA) (anxA5-DTPA) for positron emission tomography (PET) and single-photon emission computed tomography (SPECT) imaging with a single purification step. The use of a recombinant annexin A5 (cys-anxA5) with a single thiol group allowed regionally specific coupling, without affecting the binding domain of cys-anxA5.

**Results:**

The metal complexing capacity of anxA5-DTPA was investigated by labelling with ^111^In^3+^ and Eu^3+^. Binding of modified anxA5-DTPA to apoptotic cells was tested in competition experiments with a fluorescent anxA5 derivative (anxA5-FITC) using flow cytometry and compared with that of wildtype anxA5 or non-binding anxA5-DTPA (M1234-anxA5-DTPA). The binding affinity to apoptotic cells of the anxA5-DTPA conjugate does not differ from that of wildtype anxA5.

**Conclusions:**

This two-step synthesis of annexin A5-DTPA resulted in biologically active anxA5-DTPA, which can be labelled with radionuclides for use in SPECT and PET imaging.

## Background

Targeting of externalised phosphatidylserines (PS) using annexin A5 (anxA5) is relatively unspecific because it will detect not only apoptotic cells but also, for instance, necrotic cells, ageing cells, hypoxic cardiomyocytes [1], tumour blood vessels and intravascular thrombi [2]. Nevertheless, imaging using radiolabeled anxA5 can be used very sensitively and flexibly as a damage marker in a variety of clinical applications, for instance for monitoring the response to chemotherapy [3-6], where it is not important to differentiate apoptotic from necrotic cells. Moreover, it is possible to visualise purely apoptotic processes [7] with high sensitivity.

Modifications are necessary before annexin can be used as a molecular probe. This is frequently done with amine-reactive linkers (usually *N*-hydroxysuccinimide ester), which randomly react with one or more of the 21 amino groups localised at lysines in anxA5. Since it has been shown that this modification reduces the binding activity of annexin [8], and also, that all four domains present in anxA5 are necessary for optimal binding affinity [9], it is important to ensure that the binding properties of anxA5 are not impaired by these reactions.

In the present study, we use the so-called cys-anxA5, a recombinant variant of anxA5 [10], which enables selective coupling with *N*-substituted maleimides. Cys-anxA5 is derived from human anxA5 by replacing a glutamine in position 2 of the protein with cysteine and a cysteine in position 315 with serine [10]. This modification provides the protein with reactive thiol functionality, which is available for selective coupling chemistry. At pH 7, the electron-poor double bond of maleimides reacts 1,000 times more readily with thiols than with amino groups (Michael reaction). Since the thiol group is situated in the concave side of the protein, this modification is unlikely to affect the convex side of the protein which contains the PS-binding surface, and therefore, unlikely to affect the affinity of the protein for apoptotic cells. To synthesise a control probe, we used non-binding M1234-cys-anxA5 (inactive, older notation: M1M2M3M4), which has mutated calcium-binding domains and does not bind externalised PS on apoptotic cell membranes [11].

## Methods

### Chemicals

*N*-(2-aminoethyl) maleimide trifluoroacetate salt, diethylenetriamine-pentaacetic dianhydride (DTPA-DA), ethylenediaminetetraacetic acid (EDTA), dithiothreitol (DTT), anxA5-fluorescein isothiocyanate (FITC) and all other chemicals including the SDS Gel Preparation Kit were purchased from Sigma-Aldrich (Steinheim, Germany). Sodium bicarbonate was purchased from Merck (Darmstadt, Germany). The bicinchoninic acid (BCA) protein assay was purchased from Fisher Scientific GmbH (Schwerte, Germany), cys-anxA5 and M1234-cys-anxA5 from PharmaTarget (Maastricht, The Netherlands). BioGel P6 and protein standards were purchased from Bio-Rad Laboratories GmbH (Munich, Germany) and ^111^InCl_3_ solution from Covidien Deutschland GmbH (Neustadt, Germany).

### Step 1: The maleimide-DTPA linker (4)

Finely powdered *N*-(2-aminoethyl) maleimide trifluoroacetate salt **2** (29.5 μmol, 7.5 mg) was thoroughly mixed with finely powdered DTPA-DA 1 (350 μmol, 125 mg) in a 15-ml Falcon tube. The dry mixture was treated with 2 ml sodium bicarbonate solution (0.57 M, half-saturated) at 0°C (ice/water) with vigorous vortex stirring at 0°C (ice/water) for a few seconds, followed by stirring for 1 h at 0°C (ice/water) and 1 h at room temperature. The resulting yellowish solution was analysed by reversed phase high pressure liquid chromatography (HPLC). Two solvents were used for elution: Phase A (0.05% trifluoroacetic acid in water) and Phase B (0.05% trifluoroacetic acid in acetonitrile). The following gradient was applied, 5–50% B in 15 min. For electron-spray ionisation mass spectrometry (ESI-MS) the elution system was: Phase A (5 mM ammonium acetate in water) and Phase B (100% methanol) with a gradient of 0–30% B in 15 min. The flow rate was 1.0 ml/min on a Dionex-Ultimate 3000 system using a PDA-100 Photodiode Array Detector (Dionex GmbH, Idstein, Germany) with a Acclaim 120 C18 (250 mm × 4.6 mm, 5 μm, 120 Å) column. Samples were maintained at 25°C and observed at 300 nm. Peak identification was confirmed with ESI-MS (API 2000 LC/MS/MS System, Applied Biosystems, Foster City, CA, USA).

### Step 2: Preparation of cys-anxA5-DTPA (6)

#### *Typical procedure*

Potential dimers of cys-anxA5, 5 protein (9.4 mg/ml) were reduced by incubation with DTT (10 mM final concentration) for 90 min at 37°C. To remove impurities and excess DTT, the solution of reduced cys-anxA5, 5 was dialyzed for 24 h at 4°C (25 mM 4-(2-hydroxyethyl)-1-piperazineethanesulfonic acid (HEPES), 140 mM NaCl, 1 mM EDTA, pH 7.4). The resulting protein concentration was determined by BCA protein assay.

The 60 μl solution of cys-anxA5, 5 (7.2 mg/mL, 0.012 μmol) was added to 10 μl of solution of Step 1, which corresponds to eight equivalents of maleimide moieties. The mixture was incubated for 40 min at room temperature and purified by two rounds of gel filtration using a spin column with P6 gel (Bio-Rad, 1 ml, 700 × g, 4 min). The protein concentration of the resulting anxA5-DTPA 6 was determined by BCA protein assay.

Proteins were analysed by standard sodium dodecyl sulphate polyacrylamide gel electrophoresis (SDS-PAGE) and stained with Coomassie blue. The quantification was done with a G-Box-EF2 gel documentation system (VWR International GmbH, Darmstadt, Germany).

### Quantification of labelling with In^3+^/^111^In^3+^ mixture and europium

#### *Labelling with In*^*3+*^*/*^*111*^*In*^*3+*^*mixture*

In^3+^/^111^In^3+^ solution, 100 mg InCl_3_ was dissolved in 1 l 0.02 M HCl. An aliquot of 61.4 μl of this solution was mixed with 50 μl ^111^InCl_3_ solution (0.02 M HCl, 30 MBq, 16.2 ng indium) and 20 μl 0.2 M NH_4_OAc solution.

Forty microgram of anxA5-DTPA 6 was mixed with 25 μl of the In^3+^/^111^In^3+^ solution, and the mixture was incubated for 1 h at room temperature. Twenty microlitre of a Na_2_EDTA solution (2.69 mM) was added, and the mixture was stirred for 10 min at room temperature. The labelled protein was purified by two rounds of P6 gel filtration as described above. The radioactivity of both the columns and the filtrate was measured after each step. The protein concentration of the resulting complex of ^111^In^3+^ with anxA5-DTPA 6 was determined by BCA protein assay. Cys-anxA5, 5 treated under the same labelling conditions was used as a control.

#### *Labelling with Eu*^*3+*^

Twenty microgram of anxA5-DTPA 6 was mixed with 28 μl of EuCl_3_ solution (0.1 mM), and the solution was stirred for 1 h at room temperature. Eleven microlitre of a Na_2_EDTA solution (2.69 mM) was added, and the mixture was stirred for 20 min at room temperature. The labelled protein was purified as described above using a spin column of P6 gel two times. The concentration of Eu^3+^ was estimated by determination of the fluorescence of the europium chelate formed using an enhancer solution [12] and a calibration curve. The protein concentration of the resulting Eu-anxA5-DTPA complex was determined by BCA protein assay. The results were verified using unchanged cys-anxA5, 5 treated under the same labelling conditions.

### Cells and competition experiments

**Figure 1:**
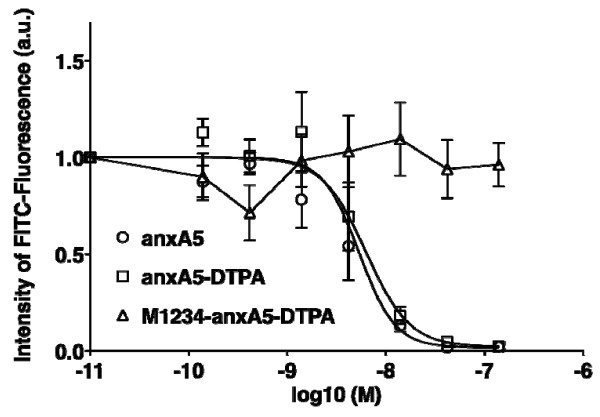
**Binding affinity of anxA5-DTPA (6).** Competition experiments demonstrate the inhibition of anxA5-FITC binding to apoptotic Jurkat T cells by active anxA5-DTPA, wildtype anxA5, and lack of inhibition by inactive M1234-anxA5-DTPA. The graph shows normalised median values of fluorescence intensity measured using flow cytometry (mean of three independent experiments ± SEM for each concentration).

Protocols were used as described before [13]. Briefly, Jurkat T cells were grown according to standard ATCC protocols. Apoptosis was induced by directly adding camptothecin to the culture medium (8 μl of 1 mM camptothecin solution per ml of culture medium) for 6 h. Cells were then washed twice in binding buffer (1.3 mM CaCl_2_, 10 mM HEPES, 150 mM NaCl, 5 mM KCl and 1 mM MgCl_2_, pH 7.4) and incubated with increasing concentrations of competitors: active anxA5-DTPA 6, inactive M1234-anxA5-DTPA and wildtype anxA5. Camptothecin-treated cells resuspended in 500 μl binding buffer (50,000 in experiments 1 and 3 and 20,000 for experiment 2) were used for each sample. After adding anxA5-FITC (a fluorescent anxA5 derivative), its binding to apoptotic cells was established from the median values of FITC fluorescence intensity in the form of the highest peaks measured by flow cytometry as described. The ability to replace anxA5-FITC on apoptotic Jurkat T cells by active anxA5-DTPA, wildtype anxA5 or inactive M1234-anxA5-DTPA was analysed by flow cytometry (FACS Calibur cytometer, Becton Dickinson GmbH, Heidelberg, Germany) according to the manufacturer's instructions. The data were fitted to dose–response curves with variable slope (Figure 1) to obtain IC50 values as evidence of displacement and the correlation coefficient using Prism 5 (GraphPad Software, Inc., San Diego, CA, USA).

### Molecular modelling

The molecular model of In-anxA5-DTPA was done using Pymol [14] software and Marvin (ChemAxon Software, Budapest, Hungary).

## Results and discussion

**Figure 2:**
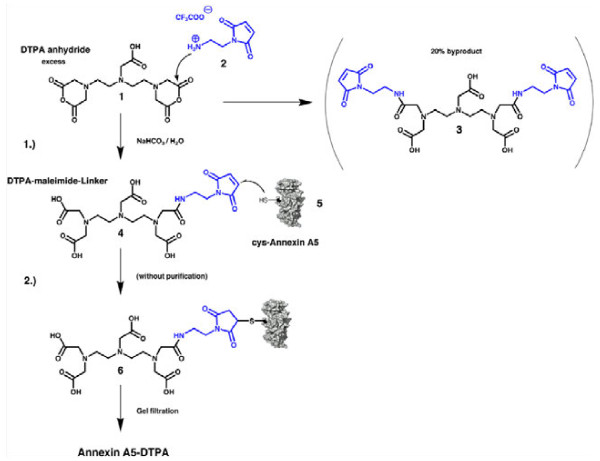
**Synthesis of anxA5-DTPA 6.** 1) Excess of cyclic DTPA anhydride 1 was reacte d with N-(2-aminoethyl) maleimide trifluoroacetate 2 in half-saturated sodium bicabonate solution. 2) Reaction product 4 was regiospecifically coupled with the thiol group of cys-anxA5, 5. Due to complete hydrolysis of excess anhydride 1 during a 2-h reaction, the reaction solution from the first step could be used directly without purification. The conjugate 6 was purified by gel filtration.

**Figure 3:**
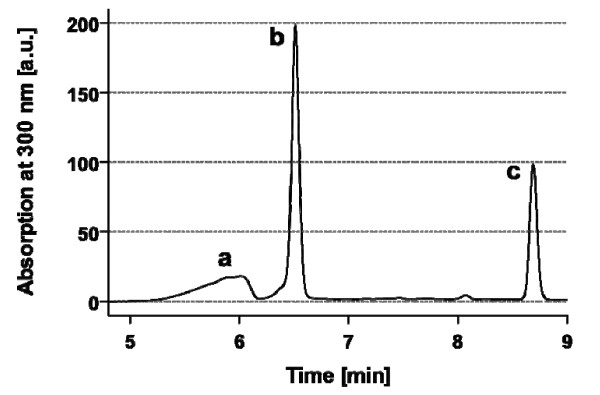
**HPLC chromatogram of the reaction between *N* -(2-aminoethyl) maleimide trifluoroacetate (2) and cyclic DTPA anhydride (1).** The peaks represent unreacted, hydrolysed DTPA (peak a), linker 4 (peak b) and byproduct 3 (peak c). Because of the presence of two UV-absorbing maleimide groups, the concentration of byproduct 3 is given by half of the peak integral in relation to peak b. The resulting ratio of product 4 to byproduct 3 is approximately 80:20.

AnxA5-DTPA 6 was synthesised in two-steps as shown in Figure 2. In the first step, cyclic DTPA anhydride 1 was reacted with *N*-(2-aminoethyl) maleimide trifluoroacetate 2 in half-saturated sodium bicarbonate solution at 0°C (ice/water). The pH of the resulting solution must not exceed 7.0 in order to prevent premature hydrolysis of the maleimide functionality. Due to the symmetry of cyclic DTPA anhydride 1, the di-addition product 3 may form, which is minimised by using a large excess of cyclic DTPA anhydride 1 compared to amine 2. The reaction conditions were optimised for maximising the yield of target linker 4 by modifying a variety of parameters such as concentration and amounts of reactants. The yield was measured by HPLC, and an optimal ratio of linker 4 to dimer linker 3 was obtained at 80:20 (mol:mol). Figure 3 shows the HPLC chromatogram of the reaction products. Peaks were assigned during the optimisation process supported by HPLC, ESI-MS and thin layer chromatography including maleimide detection with Ellman's reagent (5,5′-dithiobis-(2-nitrobenzoic acid) or DTNB) [15]. The peaks were fractionated and identified with the expected masses by ESI-MS using negative ion mode [M-H]^−^ as DTPA (m/z 392), linker 4 (m/z 514) and linker 3 (m/z 636) (Figure 3).

The resulting solution was not purified but directly reacted with cys-anxA5,5. Before reaction with the protein, any disulphide bridges must be reduced, and the reducing agent (DTT) removed by dialysis. In a second step, the linker 4 was reacted with the reduced cys-anxA5, 5, followed by purification of the protein using double gel filtration over a P6 gel. Different ratios of cys-anxA5, 5 to linker 4 were tested (2.4-fold, 8-fold and 24-fold molar excess of linker 4 over cys-anxA5, 5).

**Figure 4:**
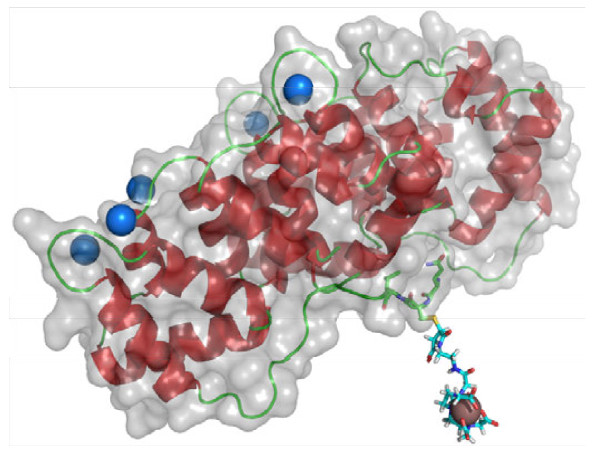
**Model of In-anxA5-DTPA.** The blue spheres are Ca^2+^ in the binding pockets at the PS-binding side of anxA5. DTPA is linked to the opposite side of the PS-binding domain of the protein. In^3+^ is represented as a brown sphere. (Crystal structure modified from Huber et al*.*[16]).

The binding capacity of the different anxA5-DTPA coupling products for trivalent metals was tested by labelling with Eu^3+^ and ^111^In^3+^. For europium labelling, the conjugate 6 was reacted with an excess of europium (III) chloride solution, and the amount of bound europium was determined after purification by measuring europium fluorescence by means of a so-called enhancer solution. The enhancer solution displaces Eu^3+^ out of the DTPA complex and ensures formation of a new, strongly fluorescent Eu^3+^ complex, from which the amount of europium can be determined using a calibration curve. Quantification by means of ^111^In^3+^ labelling was done with an excess of a mixture of cold In^3+^ and hot ^111^In^3+^ in ammonium acetate buffer with subsequent gel filtration over a P6 gel. The radioactivity persisting after two filtrations was measured and related to the amount of protein. Labelling efficiencies for metals are listed in Table 1. A model of In^3+^-labelled cys-anxA5-DTPA 6 is shown in Figure 4.

**Table 1:** **Labelling results of conjugates with Eu**^**3+**^**and**^**111**^**In**^**3+**^

**Incubation method**	^**111**^**In**^**3+**^**labelling in %**	**Eu**^**3+**^**labelling in %**
2.4-fold excess of linker 4	46.2	36.7
8-fold excess of linker 4	53.1	36.2
24-fold excess of linker 4	58.6	41.7
**for comparison:**		
cys-anxA5 5	0.0	0.0

In case of europium the yield was determined by measuring the fluorescence, in case of ^111^In^3+^ by radioactivity measurement. The numbers are percentages of labelled protein.

**Figure 5:**
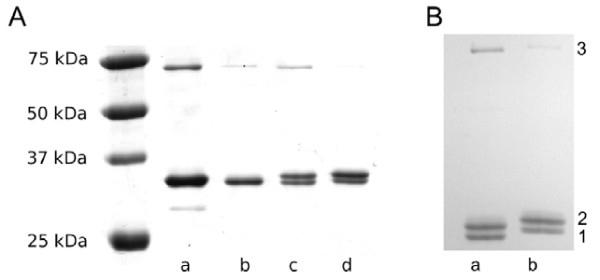
**Coomassie stained SDS-PAGE of anxA5-DTPA (6).** (**A**) The lanes show cys-anxA5, 5 (a) and M1234-anxA5 (b) in comparison to M1234-anxA5-DTPA (c) and anxA5-DTPA 6 (d) (both synthesised with eightfold excess of linker 4 in step 1). Lanes c and d show M1234-anxA5-DTPA and anxA5-DTPA 6 plus unreacted M1234-cys-anxA5 and cys-anxA5, 5. Note: No or minimal protein-linker dimers (from linker dimer 3 and cys-anxA5, 5) are visible. (**B**) Gel quantification revealed 49% yield for M1234-anxA5-DTPA (a) and 60% for anxA5-DTPA 6 (b) by dividing band 2 (DTPA derivatives) by the sum of all three bands. cys-anxA5 dimers resulting from thiol oxidation (70 kDa) are also visible (band 3).

Following optimisation of the reaction conditions, conjugates for subsequent experiments were synthesised with eightfold excess of linker 4 in step 1. For comparison, this was done with both the binding (active) form, cys-anxA5, 5 and the non-binding (inactive) form, M1234-cys-anxA5. The conjugates, anxA5-DTPA 6 and M1234-anxA5-DTPA, were examined by standard SDS-PAGE gel electrophoresis and Coomassie staining (Figure 5). The yield of anxA5-DTPA 6 and M1234-anxA5-DTPA was about 60% and 49%, respectively (Figure 5b).

### Binding of active anxA5-DTPA (6) construct to Jurkat T cells with induced apoptosis

The binding capabilities of cys-anxA5 constructs were analysed via competition measurements between active anxA5-DTPA 6 and anxA5-FITC, inactive M123-anxA5-DTPA and anxA5-FITC as well as wildtype anxA5 and anxA5-FITC for binding to apoptotic Jurkat T cells. The dose–response graph summarising three independent experiments (Figure 1) shows that active anxA5-DTPA 6 can effectively inhibit anxA5-FITC, with an IC50 of 6.2 nM (95% confidence interval 4.8 to 8.1 nM, R^2^ = 0.97), whereas wildtype anxA5 displaces anxA5-FITC with an IC50 of 5.3 nM (95% confidence interval 3.3 to 8.7 nM, R^2^ = 0.95). These results indicate that active anxA5-DTPA 6 has a similar affinity as wildtype anxA5. The inactive M1234-anxA5-DTPA was not able to inhibit anxA5-FITC binding to apoptotic Jurkat cells, as expected.

The optimised method for the direct synthesis of maleimide-DTPA linker 4 from cyclic DTPA anhydride 1 and *N*-(2-aminoethyl) maleimide trifluoroacetate 2 presented here enables single-step synthesis using inexpensive reactants. The method is straightforward to perform, and the resulting linker 4 can be purified by HPLC if needed but this is not required for the synthesis because fast hydrolysis of the anhydride prevents side reactions with amines of cys-anxA5, 5, and the resulting DTPA excess is removed by gel filtration after reaction 2 (Figure 2). Procedures for linker synthesis 4 described in the literature comprise eight [17] or five synthesis steps [18] and involve use of complex protecting group chemistry and purification steps. Subsequent reaction of the linker 4 with cys-anxA5, 5 resulted later in labelling efficiencies of 46–59% (labelling with In^3+^/^111^In^3+^ mixture), depending on the reaction conditions (Table 1). Arano et al. coupled linker 4 with an IgG antibody and found a good stability of the ^111^In^3+^-labelled conjugate during incubation for 120 h at 37°C in human serum, with about 10% loss of corrected radioactivity [17].

To preclude reactions with the amino groups of cys-anxA5, 5, linker 4 was added in excess, and the reaction time was limited to 40 min. Overall, two synthesis steps and one purification step lead to the desired conjugate 6. Based on the HPLC chromatogram, the linker dimer 3 accounts for only 20%, and there is negligible formation of protein-linker dimer from linker dimer 3 and cys-anxA5, 5 according to SDS gel electrophoresis (Figure 5). One question is whether two cys-anxA5, 5 would react with one linker dimer 3 at all or whether this is precluded for steric reasons. The coupling product that might arise in this way (linker dimer 3 with one or two cys-anxA5, 5) would have a lower affinity for the metals to be complexed [19] than anxA5-DTPA 6 due to the presence of two amides in the chelator. The yield of the DTPA coupling reaction was estimated by quantification of the protein gel electrophoresis, and dividing the band with DTPA derivatives by the sum of all bands (Figure 5b). Our coupling results are similar to yields using Cy5.5-maleimide (GE Healthcare, Solingen, Germany), and the same cys-annexins (data not shown), where complete coupling was never achieved, possibly due to steric hindrance of the cysteine of cys-anxA5.

Using the conjugate 6 prepared with an eightfold excess of linker 4, we performed competition experiments (FACS) with anxA5-FITC and apoptotic Jurkat T cells. For these experiments, a conjugate was prepared from non-binding M1234-cys-anxA5 under identical reaction conditions and compared with binding anxA5-DTPA 6 in terms of their capacity to displace anxA5-FITC. The binding capacity we measured is comparable to that of wildtype anxA5, demonstrating the advantages of second generation cys-anxA5, 5 over first generation annexin A5 [8]. The specific reaction of maleimide with the single thiol group ensures that full binding activity is retained, because the thiol group is located in the concave side of the protein whereas the four PS binding domains of anxA5 are in the convex side.

To verify the reaction of cys-anxA5, 5 with linker 4, we determined its metal chelating capacity, by labelling experiments with ^111^In^3+^ and Eu^3+^ as an additional non-radioactive method. The assumption is made that due to the high stability of such complexes, the metal binding capacity is close to the amount of DTPA coupled to cys-anxA5, 5 [17]. The protein samples labelled with an excess of metal were treated with about tenfold excess of EDTA to ^111^In^3+^ or Eu^3+^ and then gel filtered to remove excess metal and preclude nonspecific binding of the metals to the protein. The quantitative results of labelling with ^111^In^3+^ showed a better correspondence with the results of the protein gel (Figure 5b). In the case of europium labelling, Eu^3+^ was subsequently displaced from the DTPA complex by addition of an enhancer solution [12] and reacted to form a new, strongly fluorescent complex. The concentration of europium was determined using a calibration curve. This method performs very well due to the high sensitivity of fluorescence measurement but is somewhat dependent on pH [12]. Additionally the complex of Eu^3+^ with DTPA is more than six orders of magnitude weaker in comparison to In^3+^[20], which might explain the lower values compared with ^111^In^3+^-labelling. The resulting conjugate 6 can be labelled with ^99m^Tc(CO)_3_^+^[10], or ^111^In^3+^ for SPECT applications or with ^68^Ga^3+^ for PET experiments [21].

## Conclusions

In summary we demonstrated an efficient method for thiol-mediated protein labelling with DTPA, which should be applicable to other proteins including antibodies. The anxA5-DTPA 6 presented here is available as a new precursor for apoptosis imaging for *in vitro* and *in vivo* experiments that retains its PS-binding capacity due to the chemical specificity of the coupling reaction.

## Competing interests

The authors declare that they have no competing interests.

## Author's contributions

HK participated in the design, carried out the chemical syntheses and drafted the manuscript. AH did the biological characterisation. RM helped with the radioactive labelling and quantification. LS and UH helped with the HPLC and mass spectroscopy experiments. BH conceived of the study and participated in its coordination. ES designed the experiments, handled the coordination and edited the manuscript. All authors read and approved the final manuscript.
